# The role of septin 7 in physiology and pathological disease: A systematic review of current status

**DOI:** 10.1111/jcmm.13623

**Published:** 2018-03-30

**Authors:** Xinlu Wang, Fei Fei, Jie Qu, Chunyuan Li, Yuwei Li, Shiwu Zhang

**Affiliations:** ^1^ Graduate School Tianjin University of Traditional Chinese Medicine Tianjin China; ^2^ Department of Pathology Tianjin Union Medical Center Tianjin China; ^3^ Nankai University School of Medicine Nankai University Tianjin China; ^4^ Department of Colorectal Surgery Tianjin Union Medical Center Tianjin China

**Keywords:** cell proliferation and cytokinesis, filament formation, nervous and reproductive systems, septin7

## Abstract

Septins are a conserved family of cytoskeletal GTPases present in different organisms, including yeast, drosophila, *Caenorhabditis elegans* and humans. In humans, septins are involved in various cellular processes, including exocytosis, apoptosis, leukemogenesis, carcinogenesis and neurodegeneration. Septin 7 is unique out of 13 human septins. Mammalian septin 6, septin 7, septin 2 and septin 9 coisolate together in complexes to form the core unit for the generation of the septin filaments. Physiological septin filaments are hetero‐oligomeric complexes consisting of core septin hexamers and octamers. Furthermore, septin 7 plays a crucial role in cytokinesis and mitosis. Septin 7 is localized to the filopodia and branches of developing hippocampal neurons, and is the most abundant septin in the adult rat forebrain as well as a structural component of the human and mouse sperm annuli. Septin 7 is crucial to the spine morphogenesis and dendrite growth in neurons, and is also a structural constituent of the annulus in human and mouse sperm. It can suppress growth of some tumours such as glioma and papillary thyroid carcinoma. However, the molecular mechanisms of involvement of septin 7 in human disease, especially in the development of cancer, remain unclear. This review focuses on the structure, function and mechanism of septin 7 in vivo, and summarizes the role of septin 7 in cell proliferation, cytokinesis, nervous and reproductive systems, as well as the underlying molecular events linking septin 7 to various diseases, such as Alzheimer's disease, schizophrenia, neuropsychiatric systemic lupus erythematosus, tumour and so on.

## 1 INTRODUCTION

Septins were first found in budding yeast *Saccharomyces cerevisiae* as a protein family associated with cytokinesis and cell morphology.^1^ Because of the role of this protein family in the septum formation during yeast budding as well as in fungi, insects and vertebrates, they were named septins.^2^, ^3^, ^4^ Higher eukaryotic organisms have different numbers of septin isoforms ranging from 2 in *Caenorhabditis elegans*, 5 in drosophila and 13 in humans.^5^ Based on phylogenetic analysis, human septins can be divided into 4 groups (septin 2, septin 6, septin 7 and septin 9),^6^, ^7^ and 1 septin from each group can form a canonical complex^8^ to generate a number of redundant heteromeric complexes.^7^, ^8^ Septins have a unique ability to assemble into heteropolymers and form a variety of high‐order structures, including filaments, loops and cages.^9^ These unique structures can control cellular processes and localize at various cellular locations,^10^ including the plasma membrane,^11^ the annulus of spermatozoa,^12^ the bases of cilia^13^ and dendrites,^14^ as well as surrounding invasive bacteria.^15^, ^16^

Septins have been identified as the “cell‐division cycle” proteins,^17^ and they play a critical role during cytokinesis.^18^ It has been reported that septins are indispensable in co‐ordinating myosin motor proteins and bind with non‐muscle myosin II to activate myosin II in interphase and dividing cells^19^, ^20^ and reorganizingx membrane during cytokinesis,^21^ and anchoring the midbody ring structure in the membrane^22^ when a daughter cell separates from its mother cell. Septins can assemble into hetero‐oligomeric protein complexes which can further form filaments and microscopic bundles or ring structures in vitro and in vivo to control cellular processes. Septin filaments and intermediate filaments are non‐polar, distinguishing them from actin filaments and microtubules.^17^ Septins have been suggested to be cytoskeletal components owing to these structural features and their association with the membrane, F‐actin and microtubules.^8^, ^23^ Recent data indicate that they also serve as scaffolds which recruit factors to particular sites in a cell and/or act as blocks to separate different membrane areas into discrete domains to promote changes in cytoskeletal and membrane organization.^24^

The common septin structure consists of a highly variable N‐terminal domain, a central GTP‐binding domain and a C‐terminal domain, which normally includes sequences compatible with a coiled coil structure.^25^ Septin filaments are usually 7‐9 nm in width and vary in length, with unit length of 25‐32 nm observed under high salt concentration.^26^, ^27^, ^28^ G‐domains can form linear filaments using either the guanine nucleotide binding site (G interface) or N‐ and C‐terminal extensions (NC interface).^17^ Furthermore, exploring the physiological significance of GTP/GDP binding and/or GTP hydrolytic activity of septins may contribute to further understanding of their structural organization and functions.^29^

## 2 THE STRUCTURE OF THE SEPTIN 7

Septin 7, an important member of septin protein family, has an open reading frame containing 1254 nucleotides on chromosome 7P14.4‐14.1 and encodes 418 amino acids, including a GTP binding motif.^30^ cDNA sequence of septin 7 in humans is homologous to Cdc10 in yeast, and septin 7 in humans was even named hCdc10.

Three‐dimensional X‐ray structures of individual septins have shown that septin 7 shares with other septins a canonical Ras‐like G‐domain consisting of 6 ß‐strands and 5 α‐helixes.^31^ Septin 7 forms a dimer via a G interface in solution, as verified below by mutational analysis, and the monomer‐dimer equilibrium is influenced by the presence of nucleotides.^32^ Septin 7 is expected to form a septin 7‐septin 7 G interface in the polymeric form, however, the structure of the septin 7 G interface is dramatically different from the G interface of septin 2. This difference is almost entirely because of a well‐defined switch II region that was not detected in septin 2.^32^ But it is not obvious what the nature of such an interface would be and what makes septin 7 unique in the 4 human septin groups, allowing it alone among the 13 human septins to polymerize into non‐polarized filaments by occupying the ends of hexameric building blocks.

## 3 SEPTIN 7 AND CDC42 EFFECTOR PROTEINS (CDC42EPS)

Septin 7 can assemble into multimeric complexes and form filaments by combining with other septin proteins. Nevertheless, Cdc42eps can markedly alter the organization of septins within the cell, an effect that has been simultaneously discovered by the independent analysis of Cdc42‐GTP and TC10/RhoQ proteins.^33^, ^34^ Meanwhile, Cdc42‐GTP makes use of the association of Cdc42ep5 with septins to interfere with the reorganization of the septin filaments by binding to Cdc42ep5. It has been reported that the Cdc42eps are the first known negative regulators of septin reorganization providing a unique link between septins and Cdc42 GTPases. They can also be repressed by Cdc42‐GTP, a first example of the CRIB domain effect.^33^ Cdc42ep5 and Cdc42ep2 can bind septins via their BH3 domain and induce septin filament bundling.^23^, ^33^ Further characterization demonstrated that Cdc42ep5 binds specifically to septin 6/7 heterodimers or septin 2/6/7 trimers, but not to septin monomers.^35^ Using super‐resolution microscopy, it was shown that Cdc42ep3 forms an intricate filamentous network in cancer‐associated fibroblasts that colocalized with septin filaments. Budding yeast does not contain homologues of the Cdc42ep genes, indicating that the pathway that involves interaction of Cdc42eps with septin 7 has no apparent counterpart in the budding yeast.^36^ However, a similar functional linkage to septins may exist because Cdc42p deletion or mutation disrupts the septin ring structure at the yeast bud emergence site.^37^

## 4 SEPTIN 7‐ASSOCIATED COMPLEXES AND FILAMENT FORMATION

Septin family members in humans can polymerize into filamentous structures through forming homo‐ and hetero‐oligomeric complexes^29^, ^38^. Human septins are divided into the septin 2, septin 6, septin 7 and septin 9 groups. The septin 7 group seems to be unique compared to other groups, as it contains only one member in all organisms. The absence of septin 7 will lead to loss of other septin proteins in homo‐ and heterooligomeric complexes, and this protein appears essential to the generation of filaments.^8^ Abbey et al^9^ indicated that filaments are formed by alternating N‐C interfaces (formed by the interaction of N‐ and C‐termini of the septin subunits) and G‐G interfaces (formed by the interaction of the GTPase domains) between the subunits in a dual approach combining X‐ray crystallographic analysis with electron microscopy.

**Figure 1:**
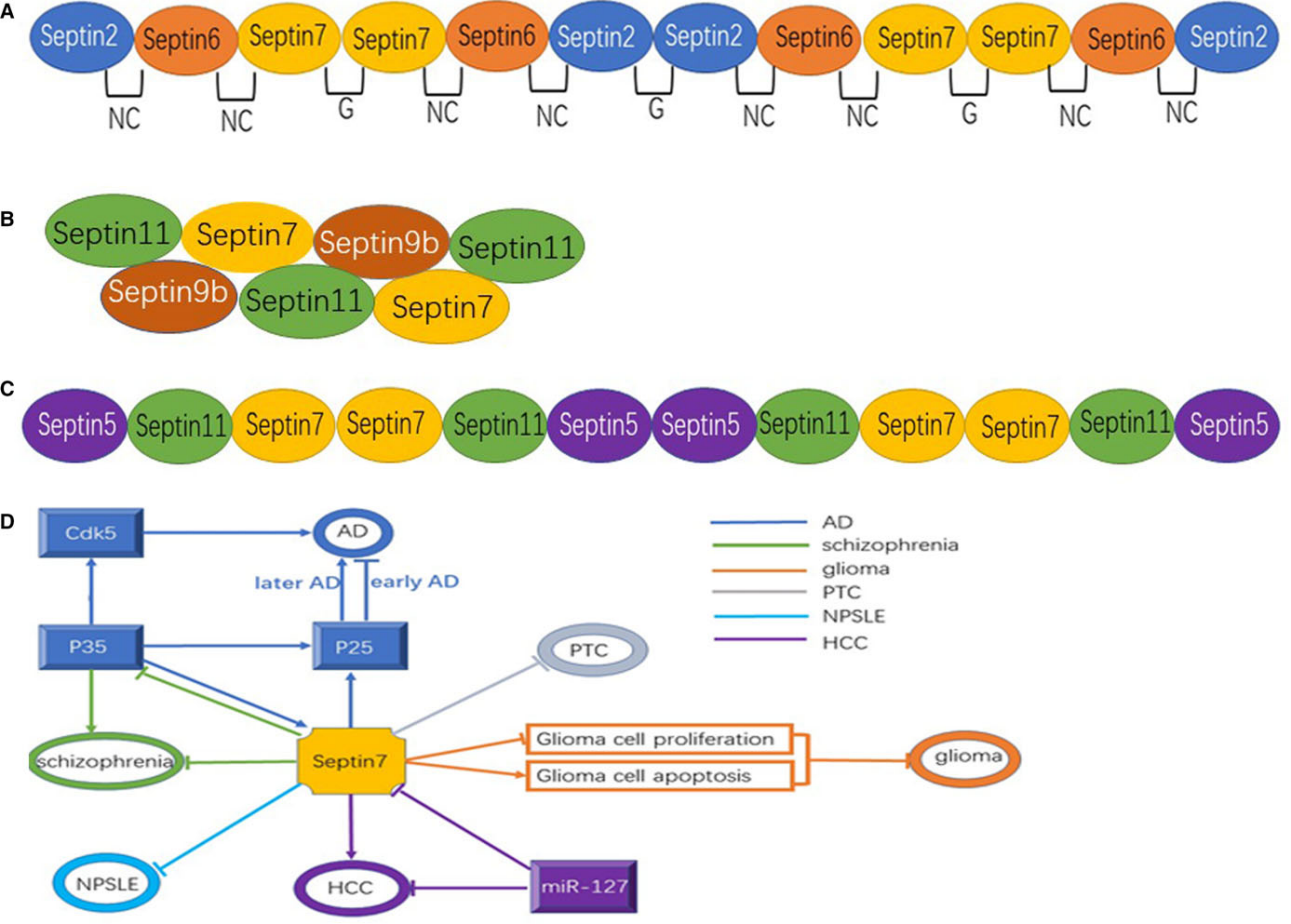
A, Organization chart of the septin2/6/7 complex. Septin 7 is a core component of septin 2/6/7. Septin 2/6/7 is the most abundant septin complex and the structure of the complex shows a universal bipolar polymer, composed of an extended G domain and/or the N‐ and C‐terminal extensions.^24^ Kinoshita et al identified that septin 2/6/7 is a non‐polar hexamer and 2 copies of each septin symmetrically arranged (septin 7/septin 6/septin 2/septin 2/septin 6/septin 7). B, Organization chart of the septin7/9b/11 complex. Septin 9b binds to C termini of both septin 7 and septin 11 through its long N‐terminal extension and septin 7/9b/11 forms a filamentous pattern along actin stress fibres in the actin filament‐dependent manner. Structure of the filaments containing septins 7/9b/11 depends on the integrity of actin filaments in REF52 cells. C, Organization chart of the septin5/7/11 complex. Septin 5 and septin 11 can colocalize and coimmunoprecipitate with septin 7. The existence of septin 5/7/11 complexes does not contradict the existence of previously reported septin 2/6/7 or septin 7/9b/11 complexes. D, Schematic Organization chart of the role of septin 7 in different diseases and the related regulation mechanism. The common structure of the septin 7 in human and other organisms and its molecular mechanism of action in physiology and disease pathology was summarized in various diseases including Alzheimer's disease (AD), schizophrenia, neuropsychiatric systemic lupus erythematosus (NPSLE), glioma, papillary thyroid carcinoma (PTC) and hepatocellular carcinoma (HCC)

Septin 7 can bind to other members of the septin family and is a core component of most multimeric septin complexes,^39^ such as septin 2/6/7,^8^, ^24^ septin 7/9b/11^29^ and septin 5/7/11.^14^, ^40^, ^41^, ^42^ Septin 2/6/7 hetero‐polymer is the only one of septin 7‐associated complexes for which a crystal structure is currently available.^31^ Septin 2/6/7 is the most abundant septin complex out of those affinity purified from brain tissues or HeLa cells.^8^ Recent analysis revealed that this heterotrimeric complex can be reconstituted in vitro. Li et al^24^ indicated that the structure of the complex shows a universal bipolar polymer, composed of an extended G domain, which forms oligomers and filaments by conserved interactions between the adjacent nucleotide binding sites and/or the N‐ and C‐terminal extensions. Kinoshita et al^8^ identified that septin 2/6/7 is a nonpolar hexamer, ~25 nm in length and ~5 nm in diameter, with 2 copies of each septin symmetrically arranged (septin 7‐septin 6‐septin 2‐septin 2‐septin 6‐septin 7) (Figure 1A). Sirajuddin et al^31^ clarified that the basic repeat unit consists of a hexamer‐septin 7:6:2:2:6:7, where septin 2‐septin 2 and septin 6‐septin 7 interactions occur via N‐C interfaces and septin 2‐septin 6 and septin 7‐septin 7 interactions occur via the G‐G interface. Septin 2/6/7 may represent a physiological complex as septin 7 provides a predominate framework for human septin complexes, and reconstituted septin complexes composed of the 3 septins are indistinguishable from the endogenous ones.^8^ Furthermore, in drosophila, dseptin 7 can form a complex with dseptin 1 and dseptin 2 in a similar fashion to human septin 7, which forms linear hexamers with septin 2 and septin 6.^43^

The in vitro septin 7‐septin 6‐septin 2‐septin 2‐septin 6‐septin 7 hexamer is an incomplete mammalian septin complex. Mammalian septin complexes can form octamers that are arranged as setpin 9‐septin 7‐septin 6‐septin 2‐septin 2‐septin 6‐septin 7‐septin 9. Septin 9 occupies the ends of an octameric mammalian septin complex.^44^ Septin 9b binds to C termini of both septin 7 and septin 11 through its long N‐terminal extension, which lacks a predicted coiled‐coil region and does not contain any predicted domain structure.^29^ Nagata et al argued that septin 7/9b/11 forms a filamentous pattern along actin stress fibres which is distributed in REF52 cells (rat embryonic fibroblast cells) in the actin filament‐dependent manner (Figure 1B). The 3 septins interact in vitro and in vivo. Structure of the filaments containing septins 7/9b/11 depends on the integrity of actin filaments in REF52 cells.^29^

Moreover, septin 5 and septin 11 can colocalize and coimmunoprecipitate with septin 7, and expression levels of both septin 5 and septin 11 decreased in septin 7‐deficient neurons.^14^, ^40^ These data suggest the existence of a septin 5/7/11 complex in neuronal dendrites, a result consistent with an earlier finding that septin 7 level is significantly decreased in homozygotic septin 5 null mice.^41^ Interestingly, several human septins might be exchangeable in septin complexes.^14^, ^40^ It has been suggested that septin 2 can be replaced by septin 5 (or septin 1/septin 4) and septin 6 by septin 11 (or possibly by septin 8/septin 10) in a septin 2/6/7 complex.^25^ Therefore, the existence of septin 5/7/11 complexes does not contradict the existence of previously reported septin 2/6/7 or septin 7/9b/11 complexes, and is consistent with earlier findings that down‐regulation of septin 7 decreased the expression of other septin complex members (Figure 1C).^8^, ^42^

Septin 7 occupies terminal positions in above mentioned hexamers,^31^ which is further associated with forming non‐polar linear septin filaments.^14^, ^17^ Septin complexes have been purified from human tissues, and some components have been identified, but mutual influences of the septins in the complexes have not been studied.

## 5 SEPTIN 7 AND INTRACELLULAR CALCIUM

Septin 7 can be regarded as a novel regulator of neuronal Ca^2+^ homoeostasis based on physiological and behavioural phenotypes.^43^ Drosophila has been identified to have 5 septin‐encoding genes^45^, ^46^, ^47^ including dseptin 7, a homologue of human septin 7. The septin 7 group is unique because it consists of a single protein both in drosophila and in humans.^17^, ^47^ Overexpression of dseptin 7 in neurons of wild‐type drosophila causes significant flight defects.^43^ Furthermore, knockdown or partial genetic depletion of dseptin 7 rescues the flight defects of animals when the reduction of inositol‐1,4,5‐trisphosphate receptor (IP3R), a protein which contributes to the release of intracellular Ca^2+^,^48^, ^49^ and the septin7 in drosophlia (dseptin7)‐deficient can compensate for the lessened function of IP3R.^43^ dseptin 7 down‐regulates the dOrai‐mediated spontaneous Ca^2+^ entry into drosophila neurons in that the dseptin7‐deficient contributes to activate the dOrai, a calcium release‐activated calcium channel protein interacting with the stromal interaction molecule (STIM) protein and other non‐canonical patterns to keep the store‐operated Ca^2+^ entry (SOCE) function.^14^, ^43^ Together, the septin7 affects the cytosolic Ca^2+^ by down‐regulating the expression of the Orai and IP3R which can cause the deficient flight ability in drosophlia. Disruption of Ca^2+^ homoeostasis has been shown to play a negative role in several neurodegenerative diseases,^43^, ^50^, ^51^ which may provide a new therapeutic target to some nervous diseases in human.

## 6 THE ROLE OF SEPTIN 7 IN THE NERVOUS SYSTEM

Septin 7 is widely distributed in the brain^39^ and has been suggested to be the most common septin in human and rat forebrain postsynaptic density (PSD) fractions by semi‐quantitative mass‐spectrometric analysis.^52^, ^53^ Moreover, the phosphorylation of septin 7 mediated by TAOK2, a gene which contributes to the spine mature,^5^ stabilizes the PSD95 in dendritic spine via its C‐terminal tail to inhibit the formation of mislocalized synapses. Furthermore the non‐phosphorylated Septin7 cannot function in the PSD95.^54^ Endogenous septin 7 is expressed in axons and clusters in dendrites of cultured hippocampal neurons localizing beneath the presynaptic membrane.^14^, ^39^ In dendritic protrusions septin 7 can form complex structures, such as an arc^8^ or gauze,^55^ similar to ring and hourglass structures formed by the yeast septin during cytokinesis.^56^, ^57^, ^58^ Interestingly, depletion of septin 7 does not decrease total protrusion density, but causes the appearance of many thin filopodia‐like protrusions. Moreover, the phenotype of septin 7 loss of function could result from the damage to the protein forming developing protrusions or may be a secondary response to the loss of mature spines.^14^

Septin 7 is crucial for regulation of dendrite branching and dendritic spine morphology. Phosphorylated septin 7 mediated by TAOK2 in the spine head facilitates to the formation and the maturation of the dendritic spine^54^. It has been identified that septin 7 is expressed at all stages of neuronal differentiation by Western blot analysis,^8^, ^59^ and redistributes and accumulates in the formation of protrusions.^60^ Septin 7 has been found at the bases of filopodia and at the branch points in developing hippocampal neurons.^54^, ^60^, ^61^ Dendrite branching can be impaired by down‐regulation of septin 7. In mature neurons, septin 7 bound to the plasma membrane and was localized at the bases of dendritic spines. Xie et al^40^ indicated that septin 7 was associated with the plasma membrane in hippocampal neurons based on significant reduction in septin 7 immunoreactivity in permeabilized hippocampal neurons after treatment with 0.2% Triton X‐100, and the down‐regulation of septin 7 disturbed the dendritic outgrowth during cell culture. Furthermore, mature septin‐deficient neurons exhibited elongated spines.^40^ Meanwhile, formation of normal branches in developing hippocampal neurons required the GTP‐binding activity of the septin 7 which may be because of GTP binding being required for septin filament polymerization, a process potentially associated with the formation of cytoskeleton.^40^ Septin 7 interacting with HDAC6 decreases the microtubule stability during the formation of the collateral in cortical neurons.^61^ In addition, septin 7 can maintain the shape of the dorsal root ganglia (DRG) neuron and their bipolar processes, meanwhile, septin 7 is required for regulating the polarity of cortical neuron rather than the DRG neuron.^60^

Overall, down‐regulation of septin 7 alters the morphology of dendritic protrusions in mature neurons.^40^ Interestingly, dendrites can be altered to form elongated protuberances, similar to the elongated buds of the septin‐deficient yeast cells.^62^ These similarities indicate that septin 7 plays a conserved function in neurons. Similar to that in yeast bud neck, it may be a key component of the dendrite diffusion barrier. This function will be a long sought after molecular association to explain the fact that dendrites are unique compartments, and that their motility is crucial for synaptic plasticity.

Alzheimer's disease (AD) is a chronic neurodegenerative disease that usually starts slowly and worsens over time.^63^ The pathological change in the AD is cerebral cortex atrophy, loss of cortical neurons, accumulation and progressive deposition of β‐amyloid in brain parenchyma and vessel wall, glial proliferation and formation of neurofibrillary tangles accompanied by the appearance of hyperphosphorylated tau.^64^ The cyclin‐dependent kinase 5 (Cdk5) is a tau kinase up‐regulated in AD,^65^ and the Cdk5 activator p35 can be cleaved to p25 to increase the Cdk5 activity.^66^ Interestingly, expression of p25 varies depending on AD stage. Reduction of p25 levels contributes to memory formation in early AD,^67^, ^68^ while the ongoing overexpression of p25 leads to neurodegeneration in later AD.^69^, ^70^ Septin 7 has been suggested to be a p25‐regulated protein localized in spine necks, where it can control the formation and shape of spines.^12^, ^38^, ^71^ Low expression of septin 7 induces lower spine density and increased size of spines in vivo.^14^, ^40^ Interestingly, septin 7 expression was specifically up‐regulated in female, but not male mice. Septin 7 can increase the spine density and reduce spine size in female p25 transgenic mice.^72^ Meanwhile, in water maze experiments septin 7 level was increased in trained mice but not in control mice, which indicates that septin 7 expression is up‐regulated during spatial memory formation.^66^ As the synapse has been identified to be affected in early AD^73^ and the association between the septin 7 and p25, investigating the role of septin 7 can have a profound effect on AD treatment.

Cdk5 signalling can alter expression of various candidate genes related to schizophrenia.^74^, ^75^, ^76^ Reduction of the level of p35, as Cdk5 activator,^66^ is sufficient to reduce septin 7 expression.^72^ Cdk5 activity can be regulated by the glutamatergic and dopaminergic cell signalling.^77^, ^78^, ^79^, ^80^ In addition,the dysfunction of the TAOK2 and septins are found in the neurofibrillary tangles in AD,^81^ which may offer a novel pathway to treat AD. Schizophrenia is a common and chronic psychiatric disorder with imprecise etiopathogenesis^82^ and has various symptoms, including disorders in sensory perception, cognition, emotions and behaviour, that could be induced by the glutamatergic dysfunction and dopaminergic disbalance.^83^ Septin 7 expression is changed in schizophrenia post‐mortem tissue and down‐regulated in the prefrontal cortex, but not the hippocampus, and expression of p35 and septin 7 are not altered by clozapine treatment. Clozapine is a kind of antipsychotics by regulating dopamine receptor D2 and other neurotransmitter receptors.^72^ Low expression of septin 7 leads to the abnormal spine density in the prefrontal cortex of schizophrenia patients.^84^, ^85^ Interestingly, septin 7 levels are reduced in prefrontal cortex of male mice, but not in female mice^86^ and the sex differences in the septin 7 levels in schizophrenia and AD are the complete opposites. However, the cause of the gender differences during the schizophrenia and AD is not still explored. Epigenetic dysregulation of septin 7 expression may result in the reduction of the p35 level which induces cognitive impairments in schizophrenia that would in turn influence septin 7 expression.^72^ Thus, enhancing the expression of septin 7 may offer us a novel way to cure the schizophrenia.

Neuropsychiatric systemic lupus erythematosus (NPSLE) is a type of systemic lupus erythematosus, a common autoimmune disease, and is characterized by multi‐systemic manifestations with both neurological and psychiatric symptoms.^87^ Septin 7 was detected in the sera of non‐NPSLE patients, but not in the sera of the NPSLE patients.^88^ This difference may reflect the deletion of pathogenic antibodies associated with alteration of brain tissue or the lack of regulatory antibodies required for maintaining neuroprotection,^88^ and it has been previously identified in normal brain tissue by control sera.^71^ Septin 7 is involved in neuronal microtubule stability, suggesting a role of microtubules in the pathophysiology of NPSLE.^88^ Taken together, septin 7 can be used to investigate the mechanism of the NPSLE.

## 7 THE ROLE OF SEPTIN 7 IN REPRODUCTIVE SYSTEM

Septin 7 is a structural constituent of the annulus of mouse and human sperm.^89^ Spermatocytes, round spermatids and elongated spermatids located in the lumen of seminiferous tubules, all can express septin 7. Also, septin 7 deficiency can cause different types of damage to the sperm.^12^, ^38^, ^90^, ^91^, ^92^ Chao et al^12^ have found the association between septin 7 and spermiogenesis: Septin 7 is widely distributed in the cytoplasm of round spermatozoa at the early stages of mouse spermatogenesis and can polymerize into a circular structure at the perinuclear area. It is also located in the caudal region of the cytoplasm which can colocalize with the mitochondria; then, the mitochondrial and septin 7 signals are shifted to the caudal part of the sperm. During the sperm tail development, the septin 7 signal becomes denser within the mitochondria in the elongated tail of the cell. At the more advanced stages of spermatogenesis, septin 7 is identified as 2 dots in the neck and annulus of the sperm with disappearance of the perinuclear ring. Finally, septin 7 is well distributed in the cytoplasm. At this stage, the proteins colocalize with mitochondria and nucleus. In mature mouse sperm, septin 7 is mainly expressed in the head, ring and, weakly, in the midpiece. Septin 7 entirely moves from the cytoplasm and extends sperm cells into the annulus in the elongated sperm cells and mature sperm.^12^

In human mature sperm, septin 7 is mainly expressed in the annulus, where it is colocalized with septin 4, and in the sperm head,^89^, ^91^ with the highest expression in the tail. The absence of the septin 7 signal often appears in the sperm with abnormal morphology and immature sperm. In the patients with asthenospermia the percentage of septin 7 deficient signals was significantly higher compared to controls, and the degree of asthenospermia appeared to be related to the percentage of defective septin 7 signals.^12^

Septin 7 may interact with δ‐tubulin during polymerization or localization of the perinuclear ring during spermatogenesis.^38^, ^90^, ^92^ Dysfunction of septin 7 may interfere with the formation of the manchette/perinuclear ring and play a negative role in resultant sperm head because of its role in formation of the perinuclear ring of the manchette.^12^ Furthermore, septin 7 expression is similar to the septin 12 expression which has been observed (as a component of the sperm annulus)^89^, ^91^ in the post‐meiotic germ cells.^38^ Septin 7/septin 12 may co‐regulate formation of all 4 subcellular compartments (acrosome, head, midpiece and tail) during spermiogenesis. In conclusion, septin 7 filaments may play a role in different intracellular diffusion events in sperm as an intracellular diffusion barrier.^12^

## 8 THE ROLE OF SEPTIN 7 IN CELL PROLIFERATION AND CYTOKINESIS

Successful cytokinesis relies on septin‐dependent and septin‐independent pathways. During septin‐dependent human cytokinesis, the presence of septin 7 is indispensable to cytokinesis for fibroblasts, but non‐essential in the hematopoietic system.^93^ Septin‐deficient T cells fail to complete cytokinesis when prompted by pharmacological activation or cytokines. Reversely, cell division is dispensable in septins when cell‐cell contacts, such as those with APCs (antigen‐presenting cells), provide a niche.^94^ Septin 7 deficiency causes embryonic lethality in early mouse embryos. Meanwhile, Menon et al^93^ indicated that septin 7‐deficient fibroblasts display incomplete cytokinesis and constitutive multinucleation by affecting mitotic spindle and midbody rather than the contractile ring. Septin 7 deficiency causes depletion of other septins but leads to near‐normal cell division in response to cues given by D10 cell lines.^95^ It is interesting to note that T cell cytokinesis in the absence of septins has also been identified in septin 7 knockout mice.^93^ Furthermore, the absence of the central subunit septin 7 did not affect the mitosis in T lymphocytes.^95^ Meanwhile, septin 7 is dispensable during the cytokinesis of myeloid cells.

Menon et al^93^ also elucidated that sufficient supplementation of stathmin could override the depletion of septin 7 and complete cytokinesis in fibroblasts, leading to a passive rescue as a result of general microtubule destabilization, and thus cytokinesis could proceed in a septin‐independent manner in the haematopoietic system. Abundant expression of stathmin in early embryos^96^ may explain the dispensability of septin up to mid‐gestation. Menon et al^93^ found that synergistic action of septins and stathmin is crucial in the completion of cytokinesis and midbody abscission. This gives us a new way to explore the mechanism of cytokinesis in vivo. Accordingly, septin 7 can be a promising target in that a solid tumour‐selective anti‐proliferative therapy against septin 7 would not damage haematopoietic cells.

Cdc10 dominates the G_1_/S transition in yeast,^10^ but its role in the cell cycle is unclear. Meanwhile, septin 7‐CENP‐E (septin 7‐centromere associated protein‐E) interaction can affect the distribution of CENP‐E for the kinetochore and chromosome alignment.^97^ Septin 7 localizes in the spindles from the pro‐MI stage to the MII stage in mouse by immunofluorescence analysis. Li et al. have found that knockdown of septin with siRNA microinjection caused high rate of formation of abnormal spindles and affected the extrusion of the first polar body. Overexpression of septin 7 hindered the alignment of chromosomes and recruitment of α‐tubulin to the spindles to affect the extrusion of the second polar body,^24^ which suggests that septin 7 plays a specific role in meiosis. In human mitosis, septin isoform may form new scaffolds in the midplane of mitotic spindles which occupy several key steps.^24^ Meanwhile, the dseptin 7 and other septins are suggested to be dispensable for the orthogonal cell division in the single‐layer neuroepithelium of the dorsal thorax except for planar cell cytokinesis.^98^ These data indicate that septin 7 plays a unique role in cytokinesis of diverse organisms.

## 9 DIFFERENT VIEWS ON SEPTIN 7 IN THE DEVELOPMENT OF CANCER

There are few reports about the role of septin 7 in cancer. To date, studies of the role of septin 7 in glioma,^99^, ^100^, ^101^, ^102^, ^103^, ^104^, ^105^ papillary thyroid carcinoma (PTC)^106^ and hepatocellular carcinoma (HCC)^107^ have been reported. In glioma and PTC, septin 7 negatively regulated the growth and progression of tumour. However, in HCC, septin 7 inhibited the growth of HCC. The opposite views about septin 7 in different kinds of cancer may be associated with the subcellular localization and post‐translational modifications of this protein.

### 9.1 Septin 7 inhibits the growth and invasion of glioma

Glioma is the most common primary malignant brain tumour, characterized by high mortality and poor prognosis.^108^ Septin 7 can suppress the growth of glioma cells by inhibiting cell proliferation and arresting the cell cycle progression at G_0_/G_1_ phase^99^ and can induce apoptosis of tumour cells.^103^ Meanwhile, depletion of septin 7 can improve glioblastoma cells migration and invasion,^103^ which lead to a proposal that septin 7 contributes to the reorganization of the actin cytoskeleton in glioblastoma cells.^100^ Expression of septin 7 in brain tumours is much lower than in normal brain tissue.^101^, ^102^ Low expression of septin 7 induces poor clinical outcomes and poor prognosis in neuroblastoma patients.^109^ Knocking down the septin 7 with siRNA in U251 xenograft tumours enhanced tumour growth compared to control tumours, and proliferation of the septin 7‐transfected U251 cells was significantly lower than that of control cells.^104^ These studies suggest that septin 7 can inhibit the growth and proliferation and induce apoptosis in glioma cells acting as a tumour‐suppressor protein. In xenograft tumours in mice treated with septin 7, proliferating cell nuclear antigen (PCNA) is down‐regulated while glial fibrillary acidic protein (GFAP) is up‐regulated.^104^ In addition, down‐regulation of Bcl‐2 and up‐regulation of caspase‐3 may indicate that septin 7 functions as a tumour suppressor in glioma.^104^ Hence, inhibition of glioma cell proliferation or promotion of apoptosis by septin 7 may be regulated by the positive or negative cell‐cycle regulators.^104^ Furthermore, up‐regulation of GFAP in TJ905 and U251 xenograft tumours treated with septin 7 indicates that septin 7 can reverse the glioma phenotypes in differentiation.^110^ Down‐regulation of MMP2/9, MTI‐MMP,^99^ integrin αvβ3 and the up‐regulation of TIMP1/2 and the redistribution of α‐tubulin after transfection with septin 7 illustrate that septin 7 inhibits migration and invasion of glioma cells^99^, ^103^. Moreover, upon overexpression septin 7 can bind to actin filaments and promote F‐actin ring formation to inhibit the migration of glioma cells.^99^ Increased levels of septin 7 promoted depolymerization of actin filaments via cofilin phospho‐regulation, and the septin 7 knockdown by cofilin phospho‐regulation improved glioma cell motility and accelerated actin polymerization. Thus, interaction of septin 7 with cofilin phosphate modulates the homoeostasis of actin and cytoskeletal motility, providing a promising candidate for new therapeutic approaches to the treatment of gliomas.^99^

MiR‐30a‐5p is a small non‐coding RNA (microRNA) that may facilitate the formation of glioma since its expression is up‐regulated in glioma cell lines and specimens.^105^ Septin 7 gene contains the highly conserved putative binding sites to miR‐30a‐5p which regulate the post‐transcriptional expression of septin 7.^105^ Septin 7 expression in control glioma cells is much lower than in glioma cells treated with miR‐30a‐5p antisense oligonucleotide. Septin 7 can be negatively regulated by miR‐30a‐5p during its translation.^105^ Furthermore, adenovirus‐mediated overexpression of septin 7 can partly reverse the increased glioma cells growth because of the down‐regulation of miR‐30a‐5p.^105^ Hence, there is an inverse correlation between septin 7 and miR‐30a‐5p, and miR‐30a‐5p decreases septin 7 expression at the translational level in glioma cells.

### 9.2 The subcellular location of septin 7 related to the development and subtype of PTC

Septin 7 is also a tumour suppressor in PTC. Papillary thyroid carcinoma is the most common form of thyroid cancer based on the histopathological differentiation of subtypes of molecular patterns into different subtypes, such as the follicular variant of PTC (FVPTC) and the classic variant of PTC (CVPTC).^111^ Expression of septin 7 and its subcellular location have been shown to be associated with specific subtypes of PTC.^111^ Nuclear, cytoplasmic and overall septin 7 expression were much lower in FVPTC tissues in contrast with benign hyperfunctioning thyroid nodules. In CVPTC group, the septin 7 expression was only decreased in the nucleus while its overall and cytoplasmic expressions were stable.^106^ The difference in septin 7 expression patterns between FVPTC and CVPTC may be associated with different molecular regulatory mechanisms and signalling pathways.^99^, ^112^

### 9.3 Septin 7 inhibits proliferation of HCC

Hepatocellular carcinoma is the primary tumour of the liver. It may result from chronic alcoholism and viral hepatitis infection. MiR‐127 level is decreased in HCC, and it reduces Huh7 cell (a hepatocellular carcinoma cell line) growth and arrests the G2/M cell cycle via suppression of septin 7 in this cell line.^107^ MiR‐127 may act as an antitumour regulator in HCC.^113^ Overexpression of MiR‐127 reduces the expression of septin 7 at its post‐transcription state in HCC tissues, and suppresses the Huh7 cell growth by down regulation of septin 7.^107^

## 10 CONCLUSION

As a highly evolutionarily conserved GTPase, septin 7 is a member of septin family which includes 13 human septins involving in exocytosis, apoptosis, leukemogenesis, carcinogenesis and neurodegeneration.^105^ Septin 7 can combine with other septins to form heteropolymers and is a core component of these multimeric septin complexes.^39^ These heteropolymers can form a diverse array of higher order structures which include filaments, gauzes and rings.^9^ However, the function and molecular mechanism of action of these heteropolymers have not received enough attention. In this review, we described the common structure of the septin 7 in human and other organisms and its molecular mechanism of action in physiology and disease pathology, summarized recent studies of the function of septin 7 in nervous and reproductive systems and showed its diverse functions in various diseases including AD, schizophrenia, NPSLE, glioma, PTC and HCC (Figure 1D). The role of septin 7 in physiology and disease pathology may provide us novel ideas for exploration of the therapeutic targets in human disease.

## CONFLICT OF INTEREST

The authors declare that there is no conflict of interest.
